# Hydrogen-Bonding-Supported Self-Healing Antifogging Thin Films

**DOI:** 10.1038/srep09227

**Published:** 2015-03-18

**Authors:** Xiaojie Zhang, Junhui He

**Affiliations:** 1 Functional Nanomaterials Laboratory, Center for Micro/Nanomaterials and Technology and Key Laboratory of Photochemical Conversion and Optoelectronic Materials, Technical Institute of Physics and Chemistry, Chinese Academy of Sciences, Zhongguancundonglu 29, Haidianqu, Beijing 100190, China; 2 University of Chinese Academy of Sciences, Beijing 100864, China

## Abstract

Inspired by the repair of DNA through efficient reformation of hydrogen bonds (H-bonds), herein we report a facile one-step approach to construction of self-healing antifogging thin films on the basis of partly cross-linked poly(vinyl alcohol)(PVA) and poly(acrylic acid)(PAA). By designing the molar ratio of hydroxyl groups to carboxyl groups, the cross-linked polymer thin films maintain abundant free hydroxyl groups to present excellent antifogging property, which is derived from the hydrophilicity and hygroscopicity of the thin films. The thin films showed smart intrinsic self-healing characteristics towards wounds caused by external forces, which is attributed to sufficient free hydroxyl groups at the scratched interfaces to reform H-bonds across the interfaces and a sufficient chain mobility that is indispensable for chain diffusion across the interfaces and hydroxyl groups association to form H-bonds. No synthetic surfaces reported so far possess all the unique characteristics of the polymer thin films: intrinsic self-healing, long-term antifogging, excellent mechanical property, high transmittance and large-scale feasibility.

It is well known that in biological systems, DNA has repair capability while tissues and organs have regenerative power. Although there is currently no analog in synthetic materials, the phenomenon of self-healing in biological systems has inspired huge research interest, and the fabrication of artificial self-healing materials has become an appealing strategy in consideration of lifetime and damage managements^1^,^2^,^3^,^4^. For example, White^5^ et al. developed a vascular repair system that can fill and polymerize a defect 10 mm in diameter and 3 mm thick. However, the integrity of repaired defects is not known, and the repaired sites do not have the same composition as the native polymer. Therefore, intrinsic self-healing is an appealing strategy of damage management compared with extrinsic self-healing. Inspired by Nepenthes, Aizenberg^6^ et al. designed a film of lubricating liquid locked in place by micro/nanoporous substrate, providing immediate self-repair through capillary wicking derived from perfluorinated fluid, which unfortunately has limits for long term operation due to the volatility of perfluorinated fluid. In nature, self-healing can occur at the level of single molecules such as the repair of DNA^7^. The formation and breaking of H-bonds between proteins and nucleic acids have been found to be a vital factor in the process of repairing oxidative damage to the purines^8^,^9^. Inspired by the repair of DNA through the efficient reformation of H-bonds, we herein report a facile one-step approach to construction of self-healing antifogging thin films based on partly cross-linked poly(vinyl alcohol)(PVA) and poly(acrylic acid)(PAA).

Owing to the design of the 3:1 ~ 1.45:1 molar ratio between hydroxyl groups and carboxyl groups, even after the esterification reaction of PVA and PAA, the polymer maintains enough free hydroxyl groups to ensure efficient reformation of H-bonds. The efficient intrinsic self-healing of the thin films is attributed to enough free hydroxyl groups at the scratched interfaces to reform H-bonds across the interfaces and a sufficient chain mobility that is indispensable for chain diffusion across the interfaces and hydroxyl groups association to form H-bonds^10^,^11^. Excellent antifogging property was observed, and is derived from the hydrophilicity and hygroscopicity of the thin films^12^. No synthetic surfaces reported so far possess all the unique characteristics of the polymer thin films: intrinsic self-healing, long-term antifogging, excellent mechanical property, high transmittance and large-scale feasibility.

The smart design and facile fabrication of the polymer thin films, which were inspired by the self-repair of the DNA damage is a breakthrough and introduction to the device of multifunctional thin films, in consideration of lifetime and damage managements. This notion would stand out as a forerunner to the materials design of our age and future.

## Results and Discussion

### Self-healing property

**Figure 1:**
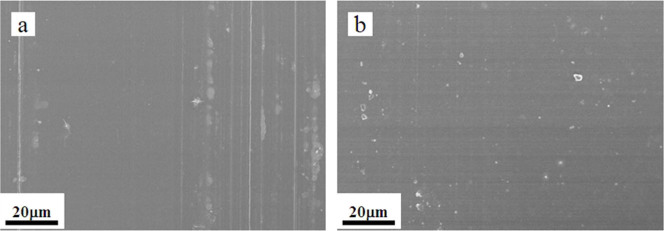
(a) SEM image of the polymer thin film after scratching with 6H pencil. The scratches are clearly visible. (b) SEM image of the scratched film surface within a few minutes under appropriate environmental temperature and humidity. The scratches have disappeared, indicating self-healing characteristics.

**Figure 2:**
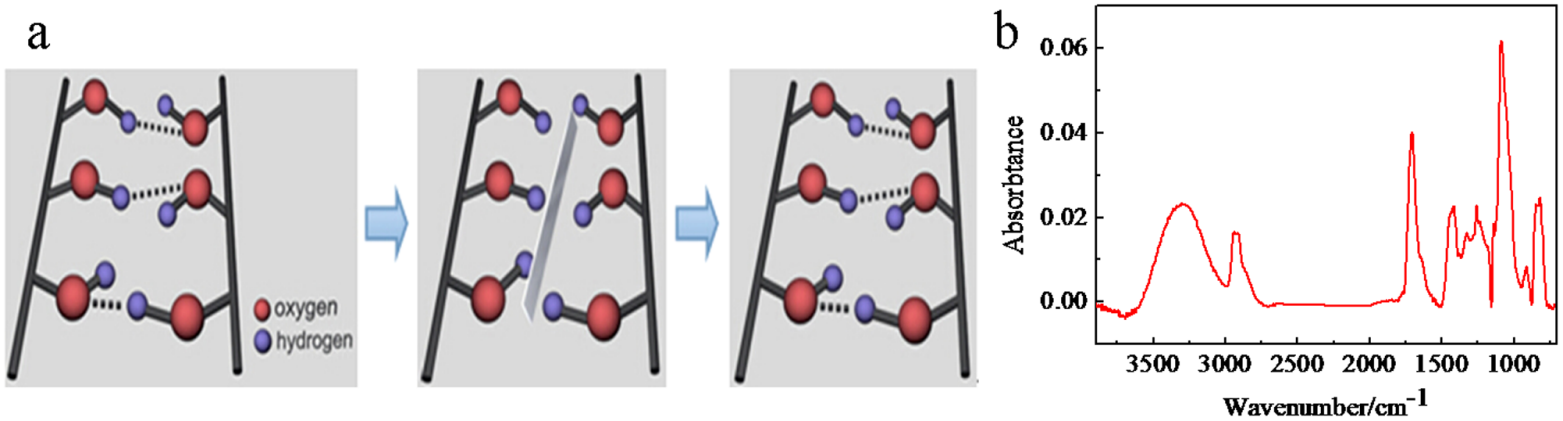
(a) Schematic illustration of the self-healing procedure, and (b) ATR-FTIR spectrum of the polymer thin film.

Thin films were deposited on substrates by one step dip-coating in an aqueous solution containing PVA and PAA with a 3:1 ~ 1.45:1 molar ratio between hydroxyl groups and carboxyl groups followed by thermal treatment at 130–150°C for 5 min. Top-view scanning electron microscopy (SEM) images show that the thin film is in fact very smooth, and scratches (Fig. 1a) created by 6H pencil could self-heal within a few minutes under appropriate environmental temperature and humidity (Fig. 1b). The efficient intrinsic self-healing of the thin film is attributed to sufficient free hydroxyl groups at the scratched interfaces to reform H-bonds across the interfaces and a sufficient chain mobility that is indispensable for chain diffusion across the interfaces and hydroxyl groups association to form H-bonds^10^,^11^, as schematically illustrated in Fig. 2a. Attenuated total reflection-Fourier transform infrared spectroscopy (ATR-FTIR)(Fig. 2b) analysis of the thin film shows two characteristic peaks, of which the broad band at 3300 cm^−1^ is attributed to the hydroxyl group, and the band at 1703 cm^−1^ originates from the carbonyl group of ester. Clearly, even after the esterification reaction, the polymer maintains sufficient free hydroxyl groups to ensure the efficient reformation of H-bonds thanks to the design of the molar ratio between hydroxyl groups and carboxyl groups.

### Antifogging property

**Figure 3:**
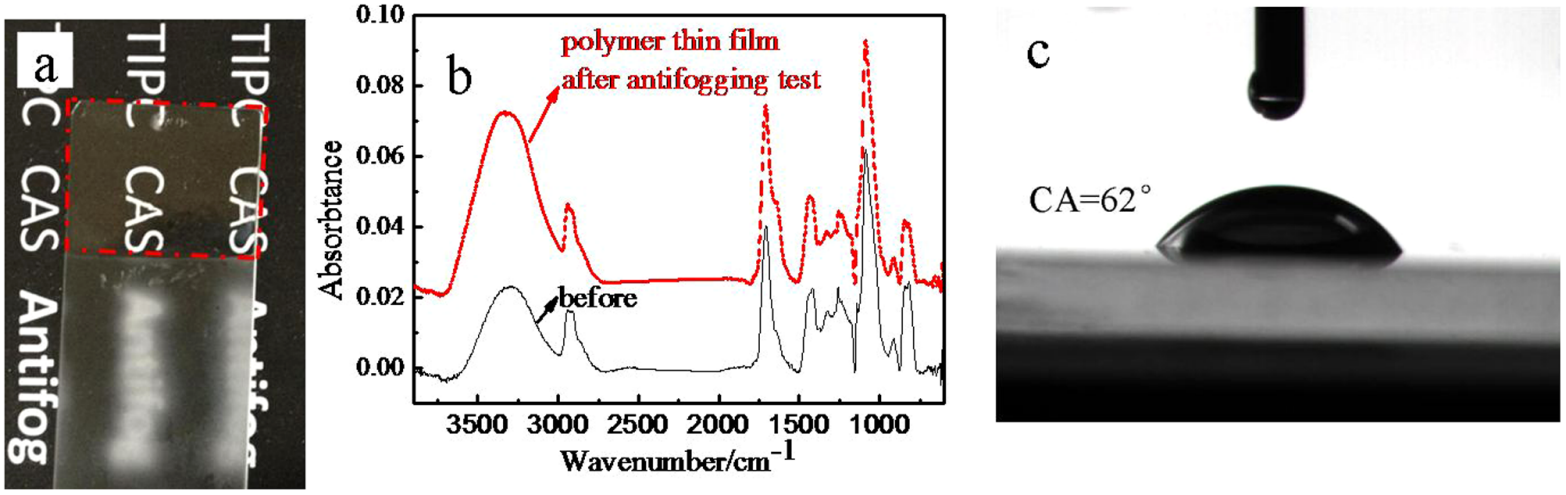
(a) The digital image exhibiting the antifogging property of polymer-coated glass (upper part) and blank glass (lower part), (b) ATR-FTIR spectra of the polymer thin film before and after antifogging test, which were normalized based on the unchanged peak intensity of –CH_2_ and (c) image of water contact angle on the polymer thin film. Water droplets of 4 μl were applied in the water contact angle measurements.

The thin film was found to exhibit excellent antifogging behavior. As shown in Figure 3a, when a substrate partly coated by the polymer was cooled at −6°C for 24 h in a refrigerator and then exposed to humid laboratory air (relative humidity (RH): 20%–40%), the uncoated part fogged immediately, and the words below were blurred by strong light scattering of tiny water droplets. In contrast, the polymer-coated part remained highly transparent, and the words below were clearly visible. The antifogging mechanism was investigated by ATR-FTIR and quartz crystal microbalance (QCM). Fig. 3b shows the ATR-FTIR spectra of the polymer-coated substrate recorded before and after the antifogging test, respectively. The ATR-FTIR spectra were normalized based on the unchanged peak intensity of –CH_2_.

The sharp increase of the peak at 3262 cm^−1^ means the increase of the amount of hydroxyl groups after the antifogging test, and the increased hydroxyl groups derive from the absorbed water by the polymer thin film preventing fogging on the polymer thin film, while the water droplets condensate on the blank glass. The absorption of water molecules must give rise to a corresponding mass change. The mass change of polymer thin film arising from the antifogging test was monitored by QCM. The QCM measurements indicate that the amount of water absorbed by the polymer (3.56 × 10^−9^ g) was 1.71 × 10^−10^ g. Therefore, the thin film weight increased by nearly 4.80 wt% because of the absorption of water molecules. According to the QCM results, if we assume one H-bond for one water molecule, the amount of H-bonds in 1 g polymer thin film is at least 2.67 × 10^−3^ mol. On the basis of the experimental results, a plausible antifogging mechanism was proposed for the polymer thin films. When water molecules in moist air from a warmer environment starts to condensate on the antifogging surface, the water molecules are rapidly absorbed into the hydrophilic domains of the polymer by hydrogen bonding and dipole-dipole interaction, preventing microdroplets from forming on the thin film surface. The absorbed water molecules may exist in the nonfreezing state^13^,^14^. According to the results on the states of water in different hydrophilic polymers by Ping^14^, in the case of PVA, the water could be kept liquid even at −100°C when the PVA concentration reaches 80% (w/w). And, our partly cross-linked PVA/PAA thin film maintains sufficient hydroxyl groups and thus the hyrophilic property. Through the interaction of water molecules and hydroxyl groups, the absorbed water by the polymer thin film must have been kept liquid at −6°C which is supported by the repeated antifogging tests. The thin film also remained transparent in the refrigerator even after absorbing water from the moist environment. And recent work by Wang^15^,^16^,^17^ also illustrated that the aqueous lubricating layer can be formed on the water swollen polymer with excellent anti-icing property.

The initial contact angle (CA) of the thin film surface was 62° (Figure 3c), demonstrating that a thin film does not have to be superhydrophilic to be effectively antifogging^12^. In the absence of long-range interfacial forces, the current understanding of wetting typically centers upon two key results: Young's law for wetting on rigid substrates and Neumann's triangle for wetting on liquid substrates. On soft solids, the total free energy includes an additional contribution from elasticity^18^,^19^,^20^. And with regard to the hygroscopicity of the polymer films, Young's law fails when there is substantial deformation near the three-phase contact line^12^.

### Optical and mechanical properties

**Figure 4:**
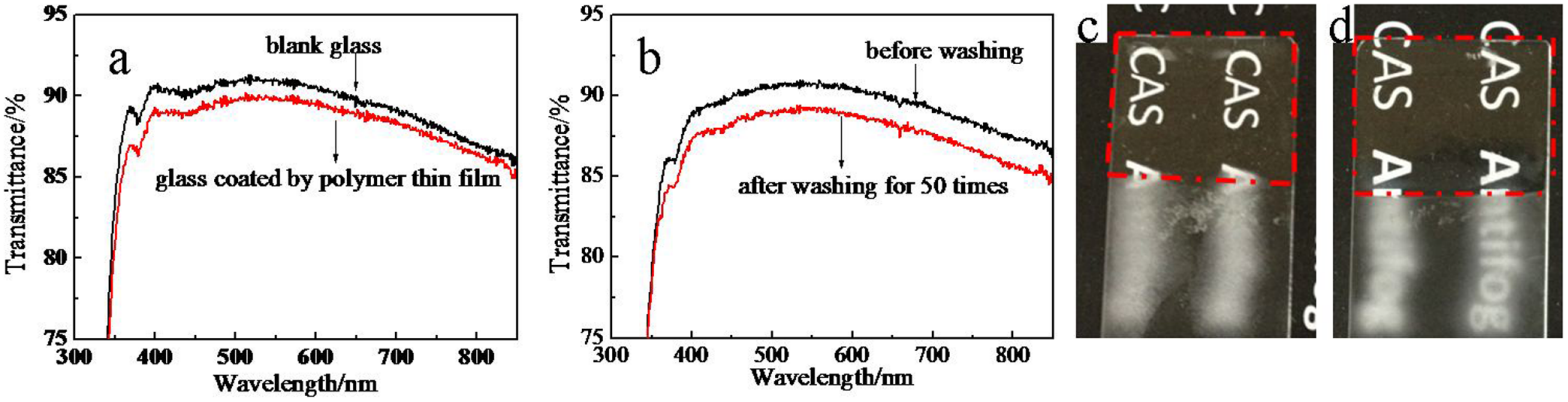
(a) Transmission spectra of blank glass and polymer-coated glass. (b) Transmission spectra of polymer-coated glass before and after 50 washing tests. The film was washed for up to 50 cycles using a sponge at a rate of 50 cycles per minute. If a film is not washed off, the thin film is believed to have good washability and can endure practical washing. (c) Digital image exhibiting antifogging property of polymer-coated glass after 50 washing tests. (d) Digital image exhibiting antifogging property of polymer-coated glass after 100 tape peeling tests. Tape peeling test was applied to examine the adhesion-to-substrate of the thin film. A tape peeling test was carried out by first pressing 3 M Scotch tape (cat. 600) on and then peeling off the thin film. One hundred peeling tests were applied.

The transmission spectra of blank glass substrate and polymer-coated glass substrate are shown in Figure 4a. The thin film only lowers the transmittance slightly as compared with blank glass substrate. Thus the thin film is highly transparent.

Mechanical properties of thin films are a key issue^21^, especially for outdoor applications. The mechanical properties of prepared thin films were assessed by washability and adhesion-to-substrate. The thin film was washed for up to 50 cycles using a sponge at a speed of 50 cycles per minute. If a thin film is not washed off, it is believed to have good washability and can endure practical washing. After washing, the thin film was kept intact only with a slight decrease in transmittance (Fig. 4b), and it also maintained the excellent antifogging property (Fig. 4c). Tape peeling test was applied to examine the adhesion-to-substrate of thin film. It was carried out by first pressing 3 M Scotch tape (cat.600) on and then peeling off the thin film. One hundred peeling tests were applied, and after the tape peeling tests, the thin film was still attached firmly to the substrate surface and maintained the excellent antifogging property (Fig. 4d). Therefore, the current thin films show excellent mechanical properties.

This polymer thin film could also be applied on flexible substrates such as poly(ethylene terephthalate)(PET). The corresponding antifogging, optical and mechanical properties are shown in the Supporting Information.

## Conclusion

DNA inspired self-healing antifogging thin films have been developed successfully. These smart polymer thin films exhibit intrinsic self-healing characteristics, outstanding long-term antifogging property, high transmittance, excellent mechanical robustness, strong adhesion to substrate and large-scale feasibility. To our best knowledge, no synthetic surfaces reported so far possess all the unique characteristics simultaneously. The criteria towards such smart polymer thin films include: (1) an appropriate molar ratio of the hydroxyl groups of PVA to the carboxyl groups of PAA in order to maintain necessary hydrophilicity and sufficient free hydroxyl groups even after esterification, and (2) cross-linking of PVA and PAA, which is essential to achieve excellent mechanical robustness and stability toward water. The current success in constructing intrinsic self-healing antifogging thin films would have broad technological implications such as eyeglasses, goggles, lenses, mirrors, and display devices in analytical and medical instruments. Doubtlessly, the smart design and facile fabrication of polymer thin films, which were inspired by the self-repair of the DNA damage, is a breakthrough and introduction to the device of multifunctional thin films in consideration of lifetime and damage managements. This notion would stand out as a forerunner to the materials design of our age and future.

## Methods

### Chemicals

Poly(vinyl alcohol)(PVA, M_n_ = 70000–90000 g/mol, 99% hydrolyzed, Aladdin) and poly (acrylic acid) (PAA, 53 wt% in water, M_w_ = 4000–7000) were purchased from Shandong Heli water treatment company. Ultrapure water with a resistivity higher than 18.2 MΩ·cm was used in all experiments, and was obtained from a three-stage Millipore Mill-Q Plus 185 purification system (Academic).

### Thin film preparation

Thin films were depoisited on substrates by one-step dip-coating followed by thermal cross-linking. First, glass and poly(ethylene terephthalate)(PET) substrates were sonicated in water for at least 10 min, and then treated with oxygen plasma (84 W, 5 min) at an oxygen flow of 800 mL·min^−1^. The substrates were then immersed in an aqueous solution containing PVA and PAA for 40 s, where the molar ratio of hydroxyl groups to carboxyl groups was set to 3:1 ~ 1.45:1 (RH: 15–40%), and withdrawn at a speed of 50 mm·min^−1^ from the solution. Finally, the thin films were thermally cross-linked at 130–150°C for 5 min.

### Characterization of thin films

The as-prepared thin films were examined by scanning electron microscopy (SEM) on a Hitachi S-4300 scanning electron microscope operated at 5 kV. Water contact angles on thin films were measured at ambient temperature on a JC2000C contact angle/interface system (Shanghai Zhongchen Digital Technique Apparatus Co.). The angle precision of which is ±0.5°. Water droplets of 4 μl were dropped carefully onto the thin film surfaces. Transmission spectra in the wavelength range of 300–900 nm were recorded using a TU-1901 spectrophotometer (Beijing Purkinje General Instrument Co.). Washability was assessed by an Elcometer 1720 Abrasion Tester. Attenuated total reflection-Fourier transform infrared spectroscopy (ATR-FTIR) analyses were carried out on a Varian Excalibur 3100 spectrometer. The mass change of the thin film arising from the antifogging test was monitored by quartz crystal microbalance (QCM). A drop-coating method was used to coat both sides (5 mm in diameter) of a silver-coated QCM resonator (9 Hz, AT-cut piezoelectric quartz crystal, Beijing Chenjing Electronics) followed by thermal cross-linking. Frequencies were recorded by Agilent 53131A universal counter linked to a computer. For the examination of antifogging property, the substrate with thin film was cooled at ca. −6°C for 24 h in a refrigerator, and then exposed to humid laboratory air (room temperature: 20–30°C, relative humidity: 20–40%).

## Author Contributions

J.H. designed the research and supervised the whole project. X.Z. designed the research, prepared samples, and performed experiments with input from J.H. J.H. and X.Z. wrote the manuscript.

## Supplementary Material

Supplementary Information Supplementary Material for Hydrogen-Bonding-Supported Self-Healing Antifogging Thin Films
