# Supplementary material for: Hydrogen-Bonding-Supported Self-Healing Antifogging Thin Films
Source: Sci Rep. 2015 Mar 18;5:9227. doi: 10.1038/srep09227 (PMC4363865; doi:10.1038/srep09227)
Supplement: Supplementary Information — Supplementary Material for Hydrogen-Bonding-Supported Self-Healing Antifogging Thin Films [file srep09227-s1.pdf]

Supplementary Material for

Hydrogen-Bonding-Supported Self-Healing Antifogging Thin Films

Xiaojie Zhang[1,2] & Junhui He[1]*

[1] Functional Nanomaterials Laboratory, Center for Micro/Nanomaterials and Technology and Key Laboratory of Photochemical Conversion and Optoelectronic Materials, Technical Institute of Physics and Chemistry, Chinese Academy of Sciences, Zhongguancundonglu 29, Haidianqu, Beijing 100190, China.

[2] University of Chinese Academy of Sciences, Beijing 100864, China.

* Correspondence and requests for material should be addressed to Junhui He (E-mail: jhhe@mail.ipc.ac.cn).

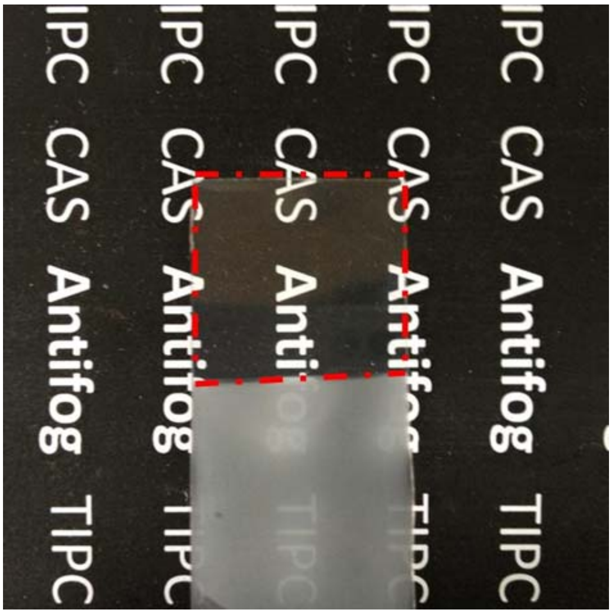

Figure S1| Digital image exhibiting antifogging property of polymer-coated PET (upper part) and blank PET (lower part).

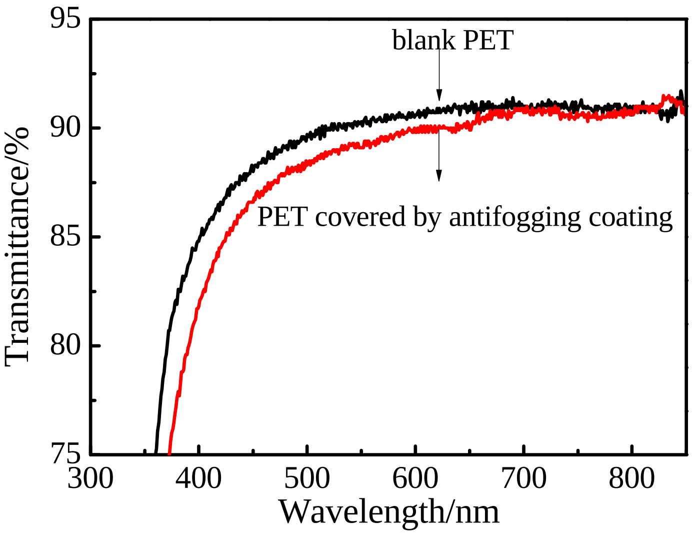

Figure S2| Transmission spectra of blank PET and polymer-coated PET.

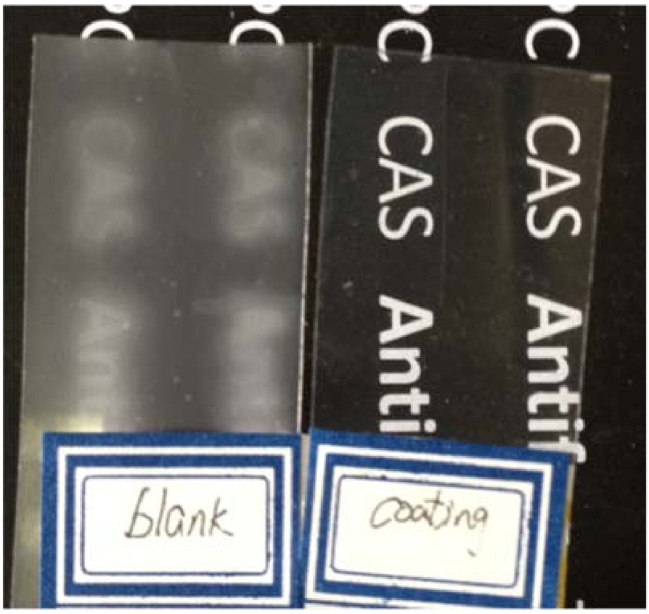

Figure S3| Digital image exhibiting antifogging property of blank PET (left) and polymer-coated PET (right) after 50 washing tests.

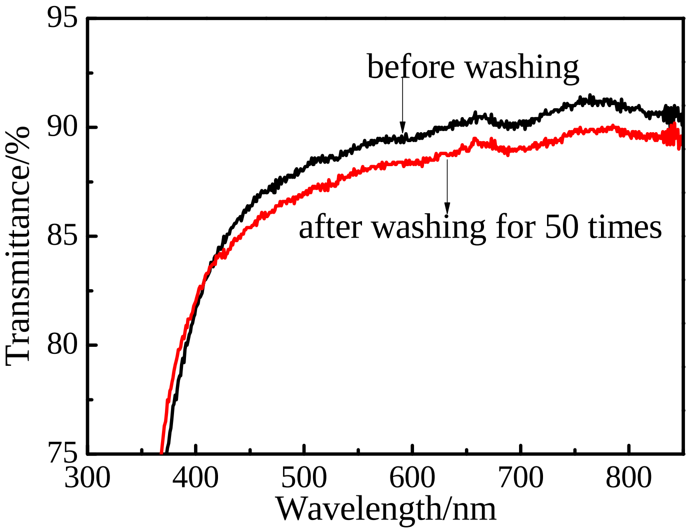

Figure S4| Transmission spectra of polymer-coated PET before and after 50 washing tests. The film was washed for up to 50 cycles using a sponge at a rate of 50 cycles per minute. If a film is not washed off, the thin film is believed to have good washability and can endure practical washing.

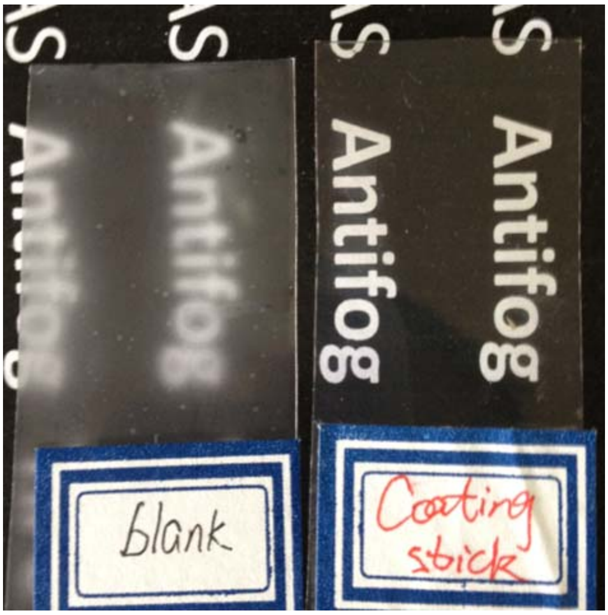

Figure S5| Digital image exhibiting antifogging property of blank PET (left) and polymer-coated PET after tape 100 peeling tests (right). Tape peeling test was applied to examine the adhesion-to-substrate of the thin film. Tape peeling test was carried out by first pressing 3M Scotch tape (cat. 600) on and then peeling off the thin film. One hundred peeling tests were applied.
